# Bipolar neutrosophic WINGS for green technology innovation

**DOI:** 10.1038/s41598-023-46699-4

**Published:** 2023-11-06

**Authors:** Yuan Tian, Kecheng Zhang

**Affiliations:** 1 School of Economics and Management, Shandong Agricultural University, Taian, China; 2 School of Business Administration, Shandong Women’s University, Jinan, China

**Keywords:** Environmental impact, Sustainability, Applied mathematics

## Abstract

Green technology innovation is a crucial assurance of achieving sustainable economic and environmental development, so improving the capability of green technology innovation is an urgent problem. In order to provide a more objective and accurate tool for identifying the most important impact factor of green technology innovation, this study innovatively proposes a new method by combining the bipolar neutrosophic sets with Weighted Influence Nonlinear Gauge System (WINGS) method. Furthermore, this paper intends to provide recommendations in improving green technology innovation capability. We invite five experts to evaluate fifteen factors influencing green technology innovation using the bipolar neutrosophic linguistic variables. Then, the proposed bipolar neutrosophic set WINGS (Bipolar NS-WINGS) method is applied to measure the influence of each impact factor of green technology innovation. Finally, we divide all the factors into cause group and effect group. Moreover, the network relation map is constructed to visualize the interrelationships between all impact factors. The Bipolar NS-WINGS suggests that Science and Technology Innovation Environment (Ω_7_) is the most important factor of green technology innovation. The result also indicates that R&D Investment (Ω_8_) is the most influential factor in which it has impacted many other factors. It is obvious that the integrated method not only enriches the research in the field of decision theory, which has not combined the bipolar-NS and WINGS method for analyzing relationships of factors, but also contributes to the improvement of green technology innovation capabilities.

## Introduction

In the context of the unstable world economic situation and the prominent ecological and environmental conflicts, green technology innovation has played an important role. Green technology innovation is receiving sustained attention because more and more people are aware of environmental sustainability^1–3^. Green innovation not only promotes economic development, but also reduces environmental damage^4^. Green technology is an important part of green innovation that can promote economic and social green development by achieving a balance between environmental protection and economic growth^5–7^. To improve green technology innovation capability, we need to figure out the factors affecting green technology innovation. It has been shown that factors such as R&D investment, environmental regulations, etc. influence the capability of green technology innovation^8–10^. For example, economic development, environmental legislation and other factors are important driving factors affecting green technology innovation, and industrial scale is the potential driving factor in the construction industry^11,12^. Digital finance can also influence the green technology innovation^13^. Researchers have used different methods to analyze the factors that influence green technology innovation. For example, Yi et al. used the Spatial Durbin Model to analyze the impact of Chinese-style fiscal decentralization on green technology innovation^14^. Li et al. utilized the data of China’s A-share listed enterprises from 2010 to 2020 to estimate the influence of government environmental punishment on green technology innovation based on the fixed-effect model^15^. And Hu et al. used the difference-in-differences model to analyze the impact of ecological civilization construction on green technology innovation^16^.

It can be seen that there are many factors that affect green technology innovation, so which is the most important one? Are there any correlations among the factors? These questions need to be addressed urgently. Unfortunately, previous studies are mainly premised on independent factors and ignores the interrelationships between the factors. Therefore, it is necessary to analyze the factors affecting green technology innovation to determine the influence of each factor and thus identify the most critical ones. It is a complex problem to study the interrelationship between the factors because of many uncertain and fuzzy variables. We can deal with this uncertainty problem with the help of fuzzy set theory. For example, Bao analyzed eight elements in the innovative governance system taking the data of agriculture-related enterprises based on the fuzzy-set qualitative comparative analysis (fsQCA)^17^. Dong et al. presented a dynamic intuitionistic fuzzy decision-making method to choose digital green innovation investment projects^18^.

Fuzzy set theory is proposed to deal with problems containing imprecise, vague, and uncertain information. By using linguistic information, it can imitate thinking and perception to deal with uncertain problems^19,20^. From the fuzzy set theory, Atanassov advanced to the intuitionistic fuzzy set with membership degree and non-membership degree^21^. The intuitionistic fuzzy set can solve problems with fuzzy information, but cannot handle the inconsistent information. Therefore, Atanassov proposed the interval-valued intuitionistic fuzzy sets to solve problems containing inconsistent information^22,23^. Then, the bipolar fuzzy set with a membership grading of [0,1] to [− 1,1] was introduced by Zhang. It satisfies not only the membership grade of [0,1], but also the membership grade of [− 1,0]^24^. According to the above theories, Smarandache proposed the neutrosophic set (NS), which has three elements of membership: truth membership (T), indeterminacy membership (I) and falsity membership (F)^25^. After that, Wang et al. proposed the single-valued neutrosophic set and the value belongs to the subset of [0,1]^26,27^. Then, Deli et al. presented the bipolar neutrosophic set (Bipolar NS)^28^. The neutrosophic set attracted a lot of attention from researchers with positive and negative membership degree. And it has been applied to solve the multi-criteria decision-making (MCDM) problems^29–32^. In recent studies, researchers combined the Bipolar NS with some operators to solve real-life MCDM problems^33–35^. For example, Jamil et al. presented bipolar neutrosophic Hamacher weighted geometric operator by combining Hamacher operators and Bipolar NS to choose a professional manager for a medicine business company^36^. Fahmi and Amin combined Bipolar NS and the prioritized muirhead mean weighted averaging operator and proposed a MCDM technique to solve problems^37^. Garai and Garg introduced a novel MCDM way based on ranking interpreter technique under single valued bipolar neutrosophic environment in order to select COVID-19 vaccines^38^.

The Decision Making Trial and Evaluation Laboratory (DEMATEL) method is one solution for dealing with the MCDM issues. It was proposed to solve difficult and complex problems and clarify the interrelationship between the factors of problems. The DEMATEL method can identify the key factors by analyzing the complex relationships between the factors using matrices or diagrams^39,40^. In addition, it can identify the causality of the factors and visualize the results by structuring casual diagram and relationship map^41^. Michnik proposed the Weighted Influence Non-linear Gauge System (WINGS) method based on DEMATEL method, which extends ability of DEMATEL and inherits all strength. Of course, it has its own unique features. Firstly, it considers not only the influence of an impact factor on other impact factors, but also the influence of that impact factor itself, while the DEMATEL only considers the former. Secondly, when the interrelations between factors are not be neglected, a special type of WINGS can be used to solve the MCDM problems^42–44^. There have been many studies combining the WINGS and other methods to solve MCDM problems. For example, Wang and Zhang integrated the grey theory and the WINGS in green supply chain management and the results of the comparison have been shown that the WINGS is more effective and suitable^45^. Wang et al. integrated WINGS method with radial basis function neural network to analyze factors of green building development^46^. Zolfani et al. introduced a new approach by combining WINGS method and the ordinal priority approach for policy making in undergraduate elective courses^47^. The WINGS method has also been used to assess vulnerability of aging levees because it can consider the strength of the vulnerability factors^48^.

In summary, this section has focused on green technology innovation, growth of bipolar NS and the WINGS. Then, a combined method integrating the bipolar NS and the WINGS is introduced to analyze the impact factors influencing the capability of green technology innovation and rank them by influence. Particularly, the innovation of this paper lies in the combination of bipolar neutrosophic sets with WINGS, which has not been combined to explain the correlation between factors in previous studies. This combination can reflect human judgment under vague and subjective conditions, consider the complex relationship between various factors and obtain accurate results. Secondly, this method can determine the importance of the influencing factors, so as to clarify the key factors. Previous studies have not ranked the influencing factors of green technology innovation ability. In addition, through the construction of causality diagram, the influencing factors are divided into cause group and effect group, and then the influence degree of each factor is analyzed. Previous studies have not carried out from this perspective. The goal of this study is applying this proposed Bipolar NS-WINGS method to identify the most important impact factor and provide recommendations in improving green technology innovation capability. By applying this method, the factors affecting green technology innovation can be ranked so that the most influential factors can be selected. And all factors are divided into causal groups to identify the causal relationships among the factors. Based on the final results, policy makers can make more effective decisions and propose measures that contribute to improving green technology innovation capabilities.

The contributions of this paper include the following aspects: (1) This paper proposes a way to decision theory combining the bipolar NS and the WINGS, which can be used to analyze complex interrelationships between factors in a vague environment. The combination of the bipolar-NS and the WINGS can enrich the research in the field of decision theory. (2) By considering the interactions between the factors of green technology innovation, this study presents a more accurate method to analyze the influence of factors. (3) It also contributes to improving green technology innovation capability, which can provide policy and management recommendations for decision makers to improve green technology innovation capability.

The following is how this study is organized. In “Definitions and arithmetic operations” section, we give some definitions of NS and bipolar NS. The proposed Bipolar NS-WINGS method is shown in “Bipolar NS-WINGS method” section. “Results” section shows how to apply the Bipolar NS-WINGS method to analyze the impact factors of green technology innovation. In “Discussion” section, we give a comparative analysis. In the last section, we give the conclusions of this study.

## Definitions and arithmetic operations

In this part, we recall some definitions and arithmetic operations of neutrosophic set and bipolar neutrosophic set.

### Neutrosophic set

#### Definition 1

^25^ Given a universe of discourse $\Psi$, $\psi$ represents a generic element in $\Psi$. Then, we can use the following equation to describe a neutrosophic set (NS) $U$ in $\Psi$.

$$U = \left\{ {\left\langle {\psi ,T_{U} (\psi ),I_{U} (\psi ),F_{U} (\psi )} \right\rangle \left| {\psi \in \Psi } \right.} \right\},$$

where $T_{U} (\psi ), I_{U} (\psi ) {\text{and}} F_{U} (\psi )$ denote the truth-membership, the indeterminacy-membership and the falsity-membership of the element $\psi \in \Psi$ to the set $U$ respectively. For each element $\psi$ in $\Psi$, there are $T_{U} (\psi ),I_{U} (\psi ),F_{U} (\psi ) \to \left[ {0,1} \right]$ and $0 \le T_{U} (\psi ) + I_{U} (\psi ) + F_{U} (\psi ) \le 3$.

### Bipolar neutrosophic set

Similar to the definition of neutrosophic set, the bipolar neutrosophic set (Bipolar NS) is presented in Definition 2.

#### Definition 2

^28^ A Bipolar NS $V$ in $\Psi$ is defined using the following equation:

$$V = \left\{ {\left\langle {\psi ,T_{V}^{ + } (\psi ),I_{V}^{ + } (\psi ),F_{V}^{ + } (\psi ),T_{V}^{ - } (\psi ),I_{V}^{ - } (\psi ),F_{V}^{ - } (\psi )} \right\rangle \left| {\psi \in \Psi } \right.} \right\},$$

where $T_{V}^{ + } (\psi ),I_{V}^{ + } (\psi ),F_{V}^{ + } (\psi ) \to \left[ {0,1} \right]{\text{ and }}T_{V}^{ - } (\psi ),I_{V}^{ - } (\psi ),F_{V}^{ - } (\psi ) \to \left[ { - 1,0} \right].$ The positive membership degree $T_{V}^{ + } (\psi ),I_{V}^{ + } (\psi ){\text{ and }}F_{V}^{ + } (\psi )$ denote the truth-membership, the indeterminacy-membership and the falsity-membership of an element $\psi \in \Psi$ associated to a Bipolar NS. Then, the negative membership degree $T_{V}^{ - } (\psi ),I_{V}^{ - } (\psi ){\text{ and }}F_{V}^{ - } (\psi )$ denote three memberships of an element $\psi \in \Psi$ to some absolute opposed quality associated to a Bipolar NS. In brief, we can use a kind of simple form $V = \left\langle {T^{ + } ,I^{ + } ,F^{ + } ,T^{ - } ,I^{ - } ,F^{ - } } \right\rangle$ to represent an element $\psi$ in a neutrosophic set, and the element $\psi$ is called a bipolar neutrosophic set number.

### Arithmetic operations of bipolar NS

The bipolar NS also fulfil some basic arithmetic rules such as inclusion, equality and so on. The rules of Bipolar NS are shown as follows.

#### Definition 3

^28^ Given two bipolar neutrosophic sets $V_{\alpha } = \left\langle {T_{\alpha }^{ + } ,I_{\alpha }^{ + } ,F_{\alpha }^{ + } ,T_{\alpha }^{ - } ,I_{\alpha }^{ - } ,F_{\alpha }^{ - } } \right\rangle$ and $V_{\beta } = \left\langle {T_{\beta }^{ + } ,I_{\beta }^{ + } ,F_{\beta }^{ + } ,T_{\beta }^{ - } ,I_{\beta }^{ - } ,F_{\beta }^{ - } } \right\rangle$. Then, some rules are shown in the following.

#### Inclusion

$V_{\alpha } \subseteq V_{\beta }$, if and only if $T_{\alpha }^{ + } \le T_{\beta }^{ + } ,I_{\alpha }^{ + } \le I_{\beta }^{ + } ,F_{\alpha }^{ + } \ge F_{\beta }^{ + } {\text{ and }}T_{\alpha }^{ - } \ge T_{\beta }^{ - } ,I_{\alpha }^{ - } \ge I_{\beta }^{ - } ,F_{\alpha }^{ - } \le F_{\beta }^{ - }$ for all $\psi \in \Psi$.

#### Equality

$V_{\alpha } = V_{\beta }$, if and only if $T_{\alpha }^{ + } = T_{\beta }^{ + } ,I_{\alpha }^{ + } = I_{\beta }^{ + } ,F_{\alpha }^{ + } = F_{\beta }^{ + } {\text{ and }}T_{\alpha }^{ - } = T_{\beta }^{ - } ,I_{\alpha }^{ - } = I_{\beta }^{ - } ,F_{\alpha }^{ - } = F_{\beta }^{ - }$ for all $\psi \in \Psi$.

#### Complement

We can use $V_{\alpha }^{c}$ to denote the complement of $V_{\alpha }$ and it is denoted as: $V_{\alpha }^{c} = \left\langle {1 - T_{\alpha }^{ + } ,1 - I_{\alpha }^{ + } ,1 - F_{\alpha }^{ + } , - 1 - T_{\alpha }^{ - } , - 1 - I_{\alpha }^{ - } , - 1 - F_{\alpha }^{ - } } \right\rangle$.

#### Union

$$V_{\alpha } \cup V_{\beta } = \left\langle {\max \left( {T_{\alpha }^{ + } ,T_{\beta }^{ + } } \right),\frac{{I_{\alpha }^{ + } + I_{\beta }^{ + } }}{2},\min \left( {F_{\alpha }^{ + } ,F_{\beta }^{ + } } \right),\min \left( {T_{\alpha }^{ - } ,T_{\beta }^{ - } } \right),\frac{{I_{\alpha }^{ - } + I_{\beta }^{ - } }}{2},\max \left( {F_{\alpha }^{ - } ,F_{\beta }^{ - } } \right)} \right\rangle .$$

#### Intersection

$$V_{\alpha } \cap V_{\beta } = \left\langle {\min \left( {T_{\alpha }^{ + } ,T_{\beta }^{ + } } \right),\frac{{I_{\alpha }^{ + } + I_{\beta }^{ + } }}{2},\max \left( {F_{\alpha }^{ + } ,F_{\beta }^{ + } } \right),\max \left( {T_{\alpha }^{ - } ,T_{\beta }^{ - } } \right),\frac{{I_{\alpha }^{ - } + I_{\beta }^{ - } }}{2},\min \left( {F_{\alpha }^{ - } ,F_{\beta }^{ - } } \right)} \right\rangle .$$

#### Addition

$$V_{\alpha } \oplus V_{\beta } = \left\langle {T_{\alpha }^{ + } + T_{\beta }^{ + } - T_{\alpha }^{ + } \cdot T_{\beta }^{ + } ,I_{\alpha }^{ + } \cdot I_{\beta }^{ + } ,F_{\alpha }^{ + } \cdot F_{\beta }^{ + } , - T_{\alpha }^{ - } \cdot T_{\beta }^{ - } , - \left( { - I_{\alpha }^{ - } - I_{\beta }^{ - } - I_{\alpha }^{ - } \cdot I_{\beta }^{ - } } \right), - \left( { - F_{\alpha }^{ - } - F_{\beta }^{ - } - F_{\alpha }^{ - } \cdot F_{\beta }^{ - } } \right)} \right\rangle .$$

#### Scalar multiplication

$$\lambda \left( {V_{\alpha } } \right) = \left\langle {1 - \left( {1 - T_{\alpha }^{ + } } \right)^{\lambda } ,\left( {I_{\alpha }^{ + } } \right)^{\lambda } ,\left( {F_{\alpha }^{ + } } \right)^{\lambda } , - \left( { - T_{\alpha }^{ - } } \right)^{\lambda } , - \left( { - I_{\alpha }^{ - } } \right)^{\lambda } , - \left( {1 - \left( {1 - \left( { - F_{\alpha }^{ - } } \right)} \right)^{\lambda } } \right)} \right\rangle \left( {\lambda > 0} \right).$$

##### Definition 4

^49^ Let $V_{\alpha } = \left\langle {T_{\alpha }^{ + } ,I_{\alpha }^{ + } ,F_{\alpha }^{ + } ,T_{\alpha }^{ - } ,I_{\alpha }^{ - } ,F_{\alpha }^{ - } } \right\rangle$ be a bipolar neutrosophic number. Then, the score function $S\left( {V_{\alpha } } \right)$ is denoted by:

$$S\left( {V_{\alpha } } \right) = \left( {T_{\alpha }^{ + } + 1 - I_{\alpha }^{ + } + 1 - F_{\alpha }^{ + } + 1 + T_{\alpha }^{ - } - I_{\alpha }^{ - } - F_{\alpha }^{ - } } \right)/6.$$

## Bipolar NS-WINGS method

**Figure 1:**
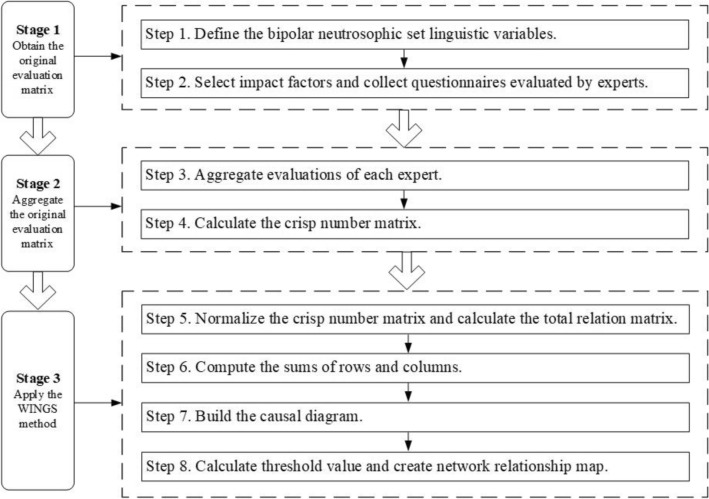
Flow chart of Bipolar NS-WINGS method.

In this section, the Bipolar NS and the WINGS method is integrated to solve specific MCDM problems. It contains three stages as in Fig. 1. Firstly, obtaining the original evaluation matrix. Secondly, the original evaluation matrix will be aggregated. Thirdly, applying the WINGS method to analyze factors. The following steps of the suggested method in detail are displayed.

### Obtain the original evaluation matrix

*Step 1* Define the bipolar neutrosophic set linguistic variables.

Firstly, we introduce the linguistic variable and they are used to determine the degree of interaction or influence between factors. Then, we propose Bipolar NS linguistic variable based on the Definition 3. It is important that the memberships in bipolar neutrosophic set must meet the requirements where $T_{V}^{ + } \left( \psi \right),I_{V}^{ + } \left( \psi \right),F_{V}^{ + } \left( \psi \right) \to \left[ {0,1} \right]{\text{ and }}T_{V}^{ - } \left( \psi \right),I_{V}^{ - } \left( \psi \right),F_{V}^{ - } \left( \psi \right) \to \left[ { - 1,0} \right]$. The linguistic variables and Bipolar NS linguistic variable are presented in Table 1.

**Table 1:** Linguistic variable and Bipolar NS linguistic variable.

Linguistic variable	Bipolar NS linguistic variable
No influence (NO)	< 0.10, 0.80, 0.90, − 0.90, − 0.80, − 0.10 >
Low influence (L)	< 0.35, 0.60, 0.70, − 0.70, − 0.60, − 0.35 >
Medium influence (M)	< 0.50, 0.60, 0.45, − 0.45, − 0.60, − 0.50 >
High influence (H)	< 0.80, 0.20, 0.15, − 0.15, − 0.20, − 0.80 >
Very high influence (VH)	< 0.90, 0.10, 0.10, − 0.10, − 0.10, − 0.90 >

*Step 2* Select impact factors and collect questionnaires evaluated by experts.

Firstly, we determine the impact factors and distribute questionnaires to experts and invite them to evaluate the factors using the five linguistic variables presented in Step 1. Suppose that there are $K$ experts evaluating $n$ impact factors. Experts are symbolized by $E_{e} = \left\{ {E_{1} ,E_{2} ,...,E_{k} } \right\}\left( {e = 1,2,...,k} \right)$, and impact factors by $\Omega_{i} = \left\{ {\Omega_{1} ,\Omega_{2} ,...,\Omega_{n} } \right\}\left( {i = 1,2,...,n} \right)$. Then, we collect evaluations of each expert and convert it into bipolar neutrosophic set linguistic variables. Let $A_{ij}^{k}$ be a bipolar neutrosophic decision matrix of the Kth experts, the original matrix is denoted by:

$$A_{ij}^{k} = \left[ {\begin{array}{*{20}c} {a_{11}^{k} } & \cdots & {a_{1n}^{k} } \\ \vdots & \ddots & \vdots \\ {a_{n1}^{k} } & \cdots & {a_{nn}^{k} } \\ \end{array} } \right],k \in K,$$

where $$a_{ij}^{k} = \left\langle {\left( {T^{ + } } \right)_{ij}^{k} ,\left( {I^{ + } } \right)_{ij}^{k} ,\left( {F^{ + } } \right)_{ij}^{k} ,\left( {T^{ - } } \right)_{ij}^{k} ,\left( {I^{ - } } \right)_{ij}^{k} ,\left( {F^{ - } } \right)_{ij}^{k} } \right\rangle ,k = 1,2,...,K;i = 1,2,...,n;j = 1,2,...,n.$$

### Aggregate the original evaluation matrix

*Step 3* Aggregate evaluations of each expert.

Let $A_{ij}$ be an aggregated decision matrix, and it is denoted by:

$$A_{ij} = \left[ {\begin{array}{*{20}c} {a_{11} } & \cdots & {a_{1n} } \\ \vdots & \ddots & \vdots \\ {a_{n1} } & \cdots & {a_{nn} } \\ \end{array} } \right],$$

where $$a_{ij} = \left\langle {T_{ij}^{ + } ,I_{ij}^{ + } ,F_{ij}^{ + } ,T_{ij}^{ - } ,I_{ij}^{ - } ,F_{ij}^{ - } } \right\rangle ,i = 1,2,...,n;j = 1,2,...,n.$$

Experts’ evaluations can be aggregated using the following equation:

1 $$a_{ij} = \left\langle {\frac{1}{K}\sum\limits_{k = 1}^{K} {\left( {T^{ + } } \right)_{ij}^{k} ,\frac{1}{K}\sum\limits_{k = 1}^{K} {\left( {I^{ + } } \right)_{ij}^{k} ,} \frac{1}{K}\sum\limits_{k = 1}^{K} {\left( {F^{ + } } \right)_{ij}^{k} ,} \frac{1}{K}\sum\limits_{k = 1}^{K} {\left( {T^{ - } } \right)_{ij}^{k} ,} \frac{1}{K}\sum\limits_{k = 1}^{K} {\left( {I^{ - } } \right)_{ij}^{k} ,} \frac{1}{K}\sum\limits_{k = 1}^{K} {\left( {F^{ - } } \right)_{ij}^{k} } } } \right\rangle .$$

*Step 4* Calculate the crisp number matrix.

The crisp number can be obtained by the following de-neutrosophication equation:

2 $$S\left( {V_{\alpha } } \right) = \left( {T_{\alpha }^{ + } + 1 - I_{\alpha }^{ + } + 1 - F_{\alpha }^{ + } + 1 + T_{\alpha }^{ - } - I_{\alpha }^{ - } - F_{\alpha }^{ - } } \right)/6.$$

After that, we can obtain a matrix containing only crisp numbers. Let $B_{ij}$ be a crisp number matrix, then the matrix is denoted as:

$$B_{ij} = \left[ {\begin{array}{*{20}c} {b_{11} } & \cdots & {b_{1n} } \\ \vdots & \ddots & \vdots \\ {b_{n1} } & \cdots & {b_{nn} } \\ \end{array} } \right]\left( {i = 1,2,...,n;j = 1,2,...,n} \right).$$

### Apply the WINGS method

*Step 5* Normalize the crisp number matrix and calculate the total relation matrix.

We can normalize the crisp number matrix by the equation:

3 $$R = A \times S,$$

where $$S = \frac{1}{{\mathop {\max }\nolimits_{1 \le i \le n} \sum\nolimits_{j = 1}^{n} {a_{ij} } }}$$.

Then, the total relation matrix can be computed by the following equation:

4 $$F = R + R^{2} + R^{3} + \cdots = \frac{R}{D - R},$$

where $D$ denotes the identity matrix and $F$ denotes the total relation matrix.

*Step 6* Compute the sums of rows and columns.

By simply adding the elements in the rows or columns of matrix $F$, we can obtain the value of $P{\text{ and }}Q$ ,which denote the sum of rows and columns respectively. The equations are defined as:

5 $$\begin{gathered} P = \sum\limits_{i = 1}^{n} {a_{ij} ,\quad i = 1,2,...,n} \hfill \\ Q = \sum\limits_{j = 1}^{n} {a_{ij} , \quad j = 1,2,...,n} . \hfill \\ \end{gathered}$$

*Step 7* Build the causal diagram.

Firstly, we compute the value of $P + Q$ and $P - Q$, which are called prominence and relation. The value of $P + Q$ indicates how significant each factor is. The value of $P - Q$ indicates the overall impact of each factor that is added to the system. In addition, $P - Q$ has positive and negative values. The positive value is referred to as net causer while the negative value is called net receiver. Then, we construct the causal diagram by horizontal axis $P + Q$ and vertical axis $P - Q$.

*Step 8* Calculate threshold value and create network relationship map.

The threshold value $\left( x \right)$ is calculated by averaging the elements in the total relation matrix $F$^50^. The element is identified as “0” if it is less than $x$, which indicates that it has less influence than other factors. On the contrary, if it is more than $x$ or equal, the element is identified as “1”, which means their influence is higher than the other factors. Next, the network relationship map is drawn to describe the connections between the impact factors in decision-making problems.

## Results

In this section, the bipolar NS-WINGS method is applied to analyze the factors of green technology innovation. The specific process and results for aggregation are shown in the following subsection.

### Data collection

*Step 1* Define the bipolar neutrosophic set linguistic variables.

The first step is introducing the linguistic variables based on the bipolar neutrosophic set. Based on the WINGS method, we define five linguistic variables firstly, then we transform them into bipolar neutrosophic set linguistic variables. Table 1 shows the linguistic variables and bipolar neutrosophic set linguistic variables.

*Step 2* Select impact factors and collect questionnaires evaluated by experts.

Firstly, we select 15 impact factors of green technology innovation with reference to previous literature^5,9,11,51^. These factors are presented in Table 2. Then, in order to collect data, we distribute questionnaires to five experts and invite them to evaluate the factors that influence green technology innovation. The details of these experts are presented in Table 3. Specifically, the experts score the degree of influence among 15 influencing factors using the five linguistic variables in Table 1. Finally, we convert it into bipolar neutrosophic set linguistic variables. One of the experts’ evaluation matrix is shown in Table 4.

**Table 2:** Impact factors for green technology innovation.

Number	Impact factors
Ω_1_	Infrastructure level
Ω_2_	Climate change
Ω_3_	Level of urbanization
Ω_4_	Human capital
Ω_5_	Market competition
Ω_6_	Government subsidies
Ω_7_	Science and Technology Innovation Environment
Ω_8_	R&D investment
Ω_9_	Industrial structure
Ω_10_	Environmental regulation
Ω_11_	Air quality
Ω_12_	Demographic structure
Ω_13_	Foreign investment
Ω_14_	Economic development
Ω_15_	Level of external opening

**Table 3:** Information of the experts.

Expert	Gender	Position	Work experience
1	Male	Professor	25
2	Female	Professor	22
3	Male	Agricultural consultant	16
4	Male	Agricultural consultant	20
5	Female	Technology manager	18

**Table 4:** Evaluation matrix of one of the experts.

	Ω_1_	Ω_2_	Ω_3_	…	Ω_15_
Ω_1_	< 0.90, 0.10, 0.10, − 0.10, − 0.10, − 0.90 >	< 0.50, 0.60, 0.45, − 0.45, − 0.60, − 0.50 >	< 0.80, 0.20, 0.15, − 0.15, − 0.20, − 0.80 >	…	< 0.80, 0.20, 0.15, − 0.15, − 0.20, − 0.80 >
Ω_2_	< 0.90, 0.10, 0.10, − 0.10, − 0.10, − 0.90 >	< 0.80, 0.20, 0.15, − 0.15, − 0.20, − 0.80 >	< 0.80, 0.20, 0.15, − 0.15, − 0.20, − 0.80 >	…	< 0.80, 0.20, 0.15, − 0.15, − 0.20, − 0.80 >
Ω_3_	< 0.90, 0.10, 0.10, − 0.10, − 0.10, − 0.90 >	< 0.90, 0.10, 0.10, − 0.10, − 0.10, − 0.90 >	< 0.90, 0.10, 0.10, − 0.10, − 0.10, − 0.90 >	…	< 0.90, 0.10, 0.10, − 0.10, − 0.10, − 0.90 >
…	…	…	…	…	…
Ω_15_	< 0.90, 0.10, 0.10, − 0.10, − 0.10, − 0.90 >	< 0.80, 0.20, 0.15, − 0.15, − 0.20, − 0.80 >	< 0.50, 0.60, 0.45, − 0.45, − 0.60, − 0.50 >	…	< 0.90, 0.10, 0.10, − 0.10, − 0.10, − 0.90 >

### Bipolar NS-WINGS analysis

*Step 3* Aggregate evaluations of each expert.

Experts’ evaluations are collected by using the Eq. (1), Table 5 presents the final results.

**Table 5:** Aggregated matrix.

	Ω_1_	Ω_2_	Ω_3_	…	Ω_15_
Ω_1_	< 0.84, 0.16, 0.13, − 0.13, − 0.16, − 0.84 >	< 0.72, 0.32, 0.25, − 0.25, − 0.32, − 0.72 >	< 0.78, 0.24, 0.19, − 0.19, − 0.24, − 0.78 >	…	< 0.64, 0.34, 0.36, − 0.36, − 0.34, − 0.64 >
Ω_2_	< 0.72, 0.32, 0.25, − 0.25, − 0.32, − 0.72 >	< 0.67, 0.34, 0.31, − 0.31, − 0.34, − 0.67 >	< 0.73, 0.26, 0.25, − 0.25, − 0.26, − 0.73 >	…	< 0.62, 0.44, 0.33, − 0.33, − 0.44, − 0.62 >
Ω_3_	< 0.74, 0.30, 0.24, − 0.24, − 0.30, − 0.74 >	< 0.77, 0.22, 0.23, − 0.23, − 0.22, − 0.77 >	< 0.79, 0.20, 0.22, − 0.22, − 0.20, − 0.79 >	…	< 0.77, 0.22, 0.23, − 0.23, − 0.22, − 0.77 >
…	…	…	…	…	…
Ω_15_	< 0.72, 0.32, 0.25, − 0.25, − 0.32, − 0.72 >	< 0.80, 0.22, 0.18, − 0.18, − 0.22, − 0.80 >	< 0.69, 0.32, 0.30, − 0.30, − 0.32, − 0.69 >	…	< 0.69, 0.32, 0.30, − 0.30, − 0.32, − 0.69 >

*Step 4* Calculate the crisp number matrix.

Table 6 shows the crisp number that is calculated using the Eq. (2).

**Table 6:** Crisp number matrix.

	Ω_1_	Ω_2_	Ω_3_	…	Ω_15_
Ω_1_	0.7367	0.6567	0.6967	…	0.5933
Ω_2_	0.6567	0.6200	0.6600	…	0.5967
Ω_3_	0.6667	0.6800	0.6900	…	0.6800
Ω_4_	0.5100	0.6400	0.5800	…	0.6200
Ω_5_	0.6467	0.6467	0.6467	…	0.6467
Ω_6_	0.5800	0.5033	0.5833	…	0.6667
Ω_7_	0.6400	0.6100	0.6133	…	0.5233
Ω_8_	0.6000	0.5633	0.4667	…	0.5000
Ω_9_	0.6400	0.6033	0.5900	…	0.5800
Ω_10_	0.7567	0.6000	0.5133	…	0.6467
Ω_11_	0.5900	0.5633	0.6967	…	0.4967
Ω_12_	0.4267	0.5067	0.5900	…	0.5033
Ω_13_	0.6000	0.5533	0.5600	…	0.5300
Ω_14_	0.6467	0.5900	0.5333	…	0.6467
Ω_15_	0.6567	0.7067	0.6300	…	0.6300

*Step 5* Normalize the crisp number matrix and calculate the total relation matrix.

We can use the Eq. (3) to normalize the crisp number matrix and Table 7 shows the final result.

**Table 7:** Normalized crisp number matrix.

	Ω_1_	Ω_2_	Ω_3_	…	Ω_15_
Ω_1_	0.0778	0.0693	0.0735	…	0.0626
Ω_2_	0.0693	0.0654	0.0697	…	0.0630
Ω_3_	0.0704	0.0718	0.0728	…	0.0718
Ω_4_	0.0538	0.0676	0.0612	…	0.0654
Ω_5_	0.0683	0.0683	0.0683	…	0.0683
Ω_6_	0.0612	0.0531	0.0616	…	0.0704
Ω_7_	0.0676	0.0644	0.0647	…	0.0552
Ω_8_	0.0633	0.0595	0.0493	…	0.0528
Ω_9_	0.0676	0.0637	0.0623	…	0.0612
Ω_10_	0.0799	0.0633	0.0542	…	0.0683
Ω_11_	0.0623	0.0595	0.0735	…	0.0524
Ω_12_	0.0450	0.0535	0.0623	…	0.0531
Ω_13_	0.0633	0.0584	0.0591	…	0.0559
Ω_14_	0.0683	0.0623	0.0563	…	0.0683
Ω_15_	0.0693	0.0746	0.0665	…	0.0665

The total relation matrix $F$ can be obtained using the Eq. (4). Table 8 shows the final results.

**Table 8:** Total relation matrix.

	Ω_1_	Ω_2_	Ω_3_	…	Ω_15_
Ω_1_	1.6048	1.5436	1.5492	…	1.5056
Ω_2_	1.6991	1.6393	1.6448	…	1.6033
Ω_3_	1.7273	1.6719	1.6741	…	1.6378
Ω_4_	1.6606	1.6194	1.6143	…	1.5842
Ω_5_	1.7412	1.6840	1.6855	…	1.6496
Ω_6_	1.7181	1.6533	1.6628	…	1.6367
Ω_7_	1.7347	1.6740	1.6761	…	1.6310
Ω_8_	1.6251	1.5675	1.5583	…	1.5289
Ω_9_	1.6375	1.5796	1.5795	…	1.5450
Ω_10_	1.7673	1.6925	1.6848	…	1.6630
Ω_11_	1.6054	1.5495	1.5647	…	1.5105
Ω_12_	1.6258	1.5801	1.5902	…	1.5475
Ω_13_	1.6926	1.6318	1.6338	…	1.5960
Ω_14_	1.7299	1.6667	1.6619	…	1.6384
Ω_15_	1.7465	1.6941	1.6875	…	1.6516

*Step 6* Compute the sums of rows and columns.

We can obtain the values of $P{\text{ and }}Q$ by summing elements rows or columns of total relation matrix $F$. Table 9 presents the values of $P,Q,P + Q{\text{ and }}P - Q$. In $P$ column, we can see that Ω_8_ has the maximum value of 26.1691, and Ω_12_ has the minimum value of 23.5466. For $P + Q$, Ω_7_ is the most significant factor with the largest value, and Ω_12_ is the least significant factor because it has the smallest value. By comparing $P + Q$, we can see the difference in the importance of each impact factor. Then, the ranking of the impact factors is obtained as Ω_7_ > Ω_10_ > Ω_6_ > Ω_8_ > Ω_13_ > Ω_14_ > Ω_3_ > Ω_5_ > Ω_15_ > Ω_2_ > Ω_11_ > Ω_1_ > Ω_4_ > Ω_9_ > Ω_12_. For $P - Q$, Ω_8_ is the most influential factor because it has the largest value and Ω_15_ has the smallest value and it is obviously influenced by the other factors.

**Table 9:** $P + Q$ and $P - Q$ for factor.

Factor	$P$	$Q$	$P + Q$	$P - Q$
Ω_1_	25.3159	23.1727	48.4886	2.1432
Ω_2_	24.4473	24.7377	49.1850	− 0.2904
Ω_3_	24.4675	25.1484	49.6159	− 0.6809
Ω_4_	24.0660	24.3942	48.4602	− 0.3282
Ω_5_	24.1679	25.3949	49.5628	− 1.2270
Ω_6_	24.7321	25.1529	49.8850	− 0.4208
Ω_7_	25.1209	25.3062	50.4271	− 0.1853
Ω_8_	26.1691	23.7086	49.8777	2.4605
Ω_9_	23.9693	23.8246	47.7939	0.1447
Ω_10_	24.3675	25.6102	49.9777	− 1.2427
Ω_11_	25.5620	23.4202	48.9822	2.1418
Ω_12_	23.5466	24.0007	47.5473	− 0.4541
Ω_13_	24.9954	24.7385	49.7339	0.2569
Ω_14_	24.4307	25.2219	49.6526	− 0.7912
Ω_15_	23.9291	25.4556	49.3847	− 1.5265

*Step 7* Build the causal diagram.

**Figure 2:**
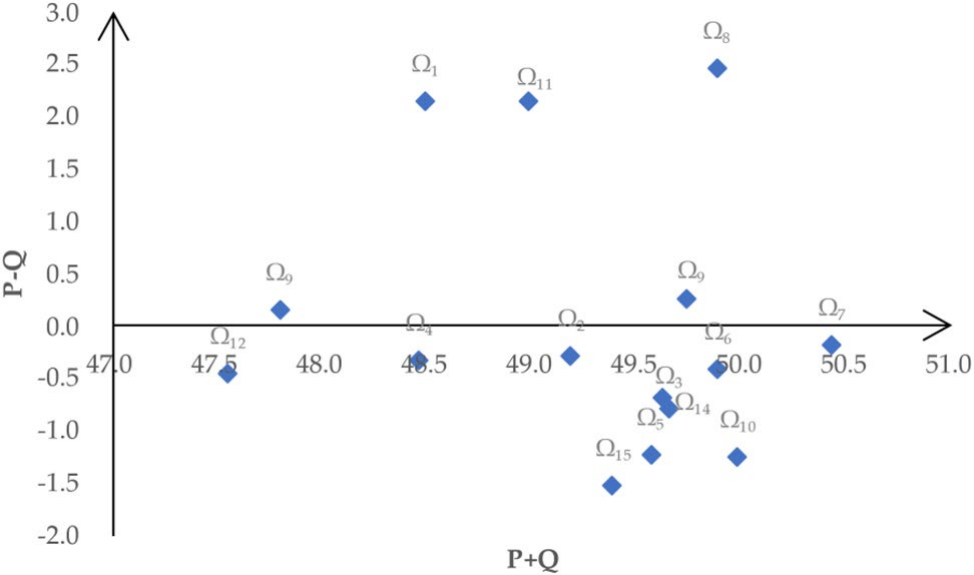
The causal diagram.

We can use horizontal axis $P + Q$ and vertical axis $P - Q$ to build the causal diagram and Fig. 2 shows the causal diagram. The horizontal axis in the figure indicates the degree of importance of each factor, we can see that Ω_7_ is at the rightmost end of the horizontal axis and it means that Ω_7_ has the greatest importance. In addition, the vertical axis shows the causal groups. According to positives and negatives of $P - Q$, which is the top and bottom of the horizontal axis, we can separate all the factors into two groups. The factors above the horizontal axis are classified as cause group while the factors below the horizontal axis are divided into effect group. In the causal diagram, we can see that the factors above the horizontal axis are Ω_8_, Ω_1_, Ω_11_, Ω_13_, and Ω_9_, which are referred to as net causer. On the contrary, factors below horizontal axis with negative values are regarded as net receiver.

*Step 8* Calculate threshold value and create network relationship map.

In total relation matrix, we can obtain the threshold value $x = 1.6413$ by calculating the average of the elements. The element is identified as “0” if it is less than $x$, which means they are less important than the other factors. The element is identified as “1” if it is more than $x$ or equal, which means they are more important than the other factors. After that, we can obtain the new total relation matrix $G$ and it is shown in Table 10.

**Table 10:** New total relation matrix.

	Ω_1_	Ω_2_	Ω_3_	Ω_4_	Ω_5_	Ω_6_	Ω_7_	Ω_8_	Ω_9_	Ω_10_	Ω_11_	Ω_12_	Ω_13_	Ω_14_	Ω_15_
Ω_1_	0	0	0	0	0	0	0	0	0	0	0	0	0	0	0
Ω_2_	1	0	1	0	0	1	1	1	0	0	1	0	1	0	0
Ω_3_	1	1	1	1	0	1	1	1	0	1	1	0	1	1	0
Ω_4_	1	0	0	0	0	0	1	1	0	0	1	0	1	0	0
Ω_5_	1	1	1	1	1	1	1	1	0	1	1	0	1	1	1
Ω_6_	1	1	1	1	1	1	1	1	0	1	1	0	1	1	0
Ω_7_	1	1	1	0	1	1	1	1	1	1	1	0	1	1	0
Ω_8_	0	0	0	0	0	0	0	1	0	0	0	0	0	0	0
Ω_9_	0	0	0	0	0	0	0	1	0	0	1	0	0	0	0
Ω_10_	1	1	1	1	1	1	1	1	1	1	1	0	1	1	1
Ω_11_	0	0	0	0	0	0	0	1	0	0	0	0	0	0	0
Ω_12_	0	0	0	0	0	0	0	1	0	0	1	0	0	0	0
Ω_13_	1	0	0	0	0	1	1	1	0	0	1	0	1	1	0
Ω_14_	1	1	1	0	1	1	1	1	1	1	1	0	1	1	0
Ω_15_	1	1	1	1	1	1	1	1	1	1	1	0	1	1	1

**Figure 3:**
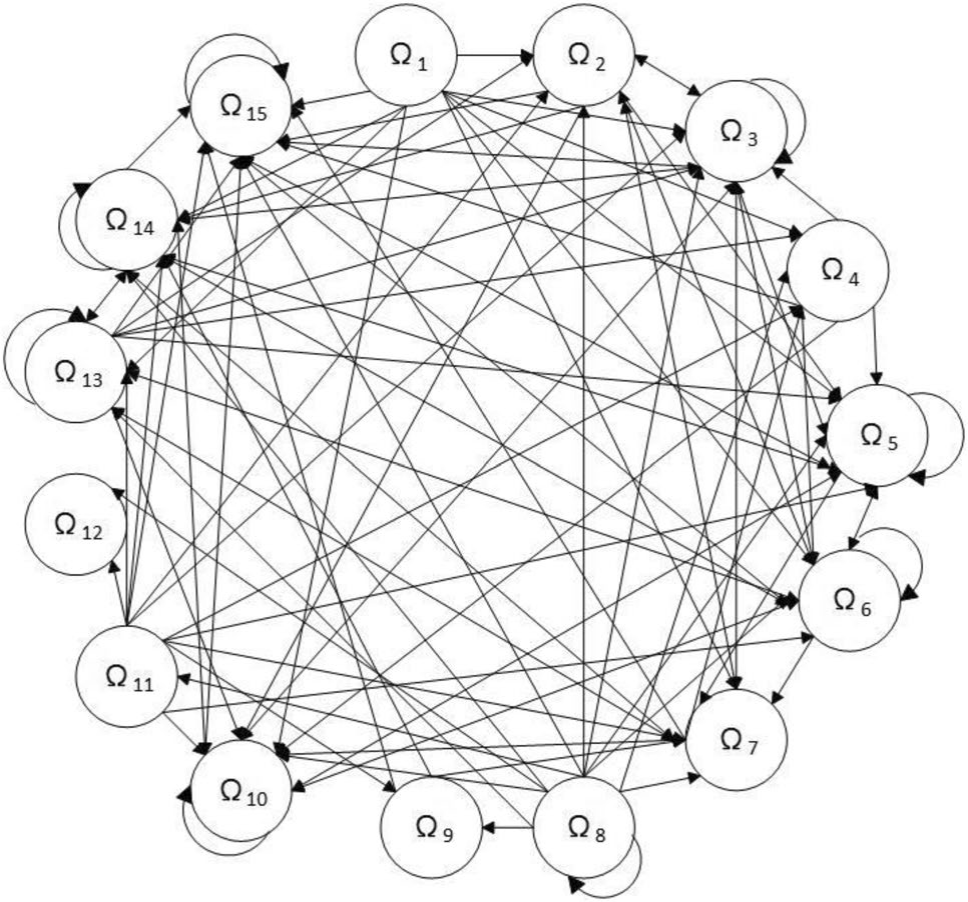
Network relationship map.

Based on the matrix $G$, we can construct the network relation map to show the direction of impact between the factors. Figure 3 shows the network relationship map and demonstrates the interrelationship between factors. For example, Ω_1_, Ω_8_ and Ω_11_ have great influence on the other factors while Ω_5_, Ω_10_ and Ω_15_ are easily influenced by the other factors and itself. We can also know that Ω_8_ is the factor that deserves the most attention because it has the greatest influence and interaction on the other factors. So it is obvious that paying more attention on Ω_8_ is helpful to improve the capability of green technology innovation.

## Discussion

In this part, the Bipolar NS-WINGS method is contrasted with WINGS method and DEMATEL method to proof the validity and reliability. Table 11 shows the specific comparative results. We can see that the ranking of three methods is similar and it can proof the feasibility of the Bipolar NS-WINGS method. However, there are some differences in the results of these three methods. For the degree of importance, the Bipolar NS-WINGS shows that the top three important factors are Science and Technology Innovation Environment (Ω_7_), Environmental Regulation (Ω_10_) and Government Subsidies (Ω_6_). The WINGS shows that the top three important factors are Science and Technology Innovation Environment (Ω_7_), Environmental Regulation (Ω_10_) and Level of urbanization (Ω_3_). The DEMATEL shows that the top three important factors are Science and Technology Innovation Environment (Ω_7_), Economic Development (Ω_14_) and Environmental Regulation (Ω_10_). It is easily known that the results of three methods all show that Science and Technology Innovation Environment (Ω_7_) is the most significant factor, and the other factors are ranked differently. The reason for this difference is that the Bipolar-NS WINGS can consider not only the interaction between factors, but also the influence of the factors themselves, which is closer to the real situation. For the cause and effect groups, three methods demonstrate a different result. The WINGS and DEMATEL have the same result, while the cause group of the Bipolar NS-WINGS method has one less factor than the other two methods. The reason for this difference is that the Bipolar-NS WINGS combines the bipolar neutrosophic set, which is beneficial to handle vague and uncertain information.

**Table 11:** Comparative results.

Method	Results
Bipolar NS-WINGS	Degree of importance Ω_7_ > Ω_10_ > Ω_6_ > Ω_8_ > Ω_13_ > Ω_14_ > Ω_3_ > Ω_5_ > Ω_15_ > Ω_2_ > Ω_11_ > Ω_1_ > Ω_4_ > Ω_9_ > Ω_12_ Net causer: Ω_8_, Ω_1_, Ω_11_, Ω_13_, Ω_9_ Net receiver:Ω_7_, Ω_2_, Ω_4_, Ω_6_, Ω_12_, Ω_3_, Ω_14_, Ω_5_, Ω_10_, Ω_15_
WINGS	Degree of importance Ω_7_ > Ω_10_ > Ω_3_ > Ω_14_ > Ω_13_ > Ω_8_ > Ω_6_ > Ω_15_ > Ω_2_ > Ω_5_ > Ω_11_ > Ω_1_ > Ω_9_ > Ω_4_ > Ω_12_ Net causer: Ω_1_, Ω_8_, Ω_11_, Ω_9_, Ω_2_, Ω_13_ Net receiver:Ω_4_, Ω_6,_ Ω_3_, Ω_12_, Ω_7_, Ω_14_, Ω_5_, Ω_10_, Ω_15_
DEMATEL	Degree of importance Ω_7_ > Ω_14_ > Ω_10_ > Ω_6_ > Ω_13_ > Ω_3_ > Ω_15_ > Ω_2_ > Ω_8_ > Ω_11_ > Ω_5_ > Ω_12_ > Ω_1_ > Ω_4_ > Ω_9_ Net causer: Ω_1_, Ω_8_, Ω_11_, Ω_9_, Ω_2_, Ω_13_ Net receiver:Ω_4_, Ω_6_, Ω_3_, Ω_12_, Ω_7_, Ω_14_, Ω_5_, Ω_10_, Ω_15_

In contrast to DEMATEL, both Bipolar-NS WINGS and WINGS consider the effect of the factor itself, which is useful for studying the interrelationship between the factors. And compared to WINGS and DEMATEL, the Bipolar-NS WINGS combines the bipolar neutrosophic set, which is beneficial to integrate the opinions of different experts. Therefore, compared with other methods, the method proposed in this paper has the following advantages. Firstly, this method is based on the bipolar neutrosophic set, which can employ linguistic variables to express the expert’s tacit knowledge and help make evaluations. The main significance in application of this work is to define and develop an effective evaluation framework to guide managers in assessing the factors affecting the ability to innovate in green technologies. This method overcomes the one-sidedness of DEMATEL and WINGS, and makes the evaluation result more objective and real. In addition, the results of comparison with the other two methods confirm that the final ranking of the method is credible. Obviously, this study provides a more accurate, effective and systematic decision support tool for studying the influencing factors of green innovation capability. Secondly, using the WINGS approach enables consideration of the factors themselves and the interactions between factors. According to the final results, it can classify factors into causal group to identify cause-and-effect relationships between factors. The results show that Technological Innovation Environment (Ω_7_) is the most important factor and R&D investment (Ω_8_) is the most influential factor, which has an impact on other factors. Therefore, managers should focus on these two factors. Increasing R&D investment and improving the technological innovation environment play an important role in improving the ability of green technology innovation. Finally, this study can help researchers to better understand the problem of green technology innovation ability in theory, and also help organizations to design and develop a better green technology innovation ability evaluation system. And the novel method can be applied in more environments where information is ambiguous and uncertain, and it has generality.

There are some limitations to this article. Firstly, the evaluation of each expert may have some prejudice and more experts may be requested to participate in this evaluation in the future. Secondly, we only considered 15 impact factors that affect green technology innovation, so there may be some other factors that are not considered. Finally, more MCDM methods can be combined with the bipolar neutrosophic set based on this paper in the future.

## Conclusions

Green technology innovation is receiving sustained attention because more and more people are aware of environmental sustainability. To improve green technology innovation capability, we need to figure out the factors affecting green technology innovation. Therefore, we propose a novel method combining bipolar neutrosophic set with WINGS method to analyze the interrelationship between factors and rank them by influence. Firstly, we define the bipolar neutrosophic set linguistic variables and select fifteen impact factors of green technology innovation. Secondly, we invite five experts to score the importance of each factor and convert it into bipolar neutrosophic set number matrix. Thirdly, we aggregate the crisp number matrix and calculate the importance of each factor by using the Bipolar NS-WINGS method. In addition, we obtain the relationship between impact factors and the causal groups of green technology innovation by applying the proposed Bipolar-NS WINGS method. This paper also shows the interrelationship between factors by constructing the network relationship map. These findings can provide some initial guidance for improving green technology innovation capabilities, and it is meaningful for studying the relative relationships among the factors affecting green technology innovation.

The final result indicates that the most important factor is Science and Technology Innovation Environment (Ω_7_). It means that Science and Technology Innovation Environment is the most important factor for improving green technology innovation capabilities. As we all know, Science and Technology Innovation Environment is the basis for innovative activities and creating a good innovation environment is the first step to improve the capabilities of green technology innovation. The support of local governments for scientific and technological innovation can effectively promote the enthusiasm of green technology research and development, guide the implementation of green innovation activities and improve the ability of green innovation. Therefore, policy makers should strive to create a favorable environment for green innovation^52^. For example, the government should continuously optimize the construction of innovation infrastructure and the structure of fiscal science spending and attract more market forces to participate in green technology innovation activities. Environmental Regulations (Ω_10_) is the second most important factor. In fact, scholars have extensively explored the effects of environmental regulations on the effectiveness of green development of urban industries, etc., and generally found that environmental regulations have the impacts of short-term inhibition and long-term promotion^53,54^. Some studies have shown that the excessive growth of environmental regulation level will inhibit green innovation, so it is necessary to properly control the level of environmental regulation. Different types of environmental regulations may have different impacts on green technology innovation. Therefore, the government should formulate reasonable environmental regulation standards.

Government subsidies (Ω_6_) is also an important factor. The development of innovation activities requires a large amount of capital investment. General scholars believe that the degree of government support for enterprise innovation activities can make up for the lack of investment in enterprise green technology innovation, and then have a positive effect on enterprise green technology innovation. Therefore, the government should consider increasing research more subsidies to improve green technology innovation capabilities. Foreign investment (Ω_13_) will intensify local market competition, and then have a certain incentive effect on industrial enterprises, forcing local enterprises to increase research and development investment to improve the level of technology. Economic Development (Ω_14_) and Level of urbanization (Ω_3_) are also important factors affecting the ability of green technology innovation. Generally speaking, the higher the level of regional economic development, the greater the protection of the environment and the more urgent the need for green development. The high level of urbanization often indicates a more developed level of local economic development, a more complete urban management system, and a stronger attraction for professional and technical talents, which plays a positive role in promoting local governments and enterprises to carry out green technology innovation activities and improve green technology innovation capabilities.

This study also notes that R&D Investment (Ω_8_) is the most influential factor in which it has impacted many other factors. R&D investment, as a form of expression of technological level, directly determines the output results of the region. Rational use of R&D funds, precise investment, and clear innovation types can promote the coordinated development of economy and environment and improve the ability of green technology innovation. Therefore, increasing R&D investment will have an impact on other factors, such as improving the R&D environment. Generally speaking, the more invested in R&D, the easier it is to conduct innovative activities, then the level of technological capability is higher^55^. Moreover, according to the value of $P - Q$, we divide all the factors into two groups. Infrastructure Level (Ω_1_), R&D Investment (Ω_8_), Industrial Structure (Ω_9_), Air Quality (Ω_11_) and Foreign Investment (Ω_13_) are gathered into cause group and it is essential to focus on these factors while other factors are divided into effect group. Therefore, policy makers should pay more attention to these factors, such as improving the level of infrastructure and increasing R&D investment, to lay the foundation for improving green technology innovation capabilities.

This paper will provide some suggestions for improving green technology innovation capabilities according to the final results. Policymakers can make policies refer to the important and influential factors. For example, it is helpful for improving green technology innovation capabilities to improve the Science and Technology Innovation Environment and increase R&D Investment. In addition, the bipolar neutrosophic set may be combined with more methods to solve more MCDM problems in the future.

### Ethical approval

All methods were carried out in accordance with relevant guidelines and regulations.

All experimental protocols were approved by the Ethics Committee of the College of Economics and Management of Shandong Agricultural University.

Informed consent was obtained from all subjects.

## Data Availability

All relevant data are within the paper.
